# Enrichment of branched chain amino acid transaminase 1 correlates with multiple biological processes and contributes to poor survival of IDH1 wild-type gliomas

**DOI:** 10.18632/aging.202328

**Published:** 2021-01-20

**Authors:** Li Yi, Xiaoguang Fan, Jiabo Li, Feng Yuan, Jian Zhao, Monica Nistér, Xuejun Yang

**Affiliations:** 1 Department of Neurosurgery, Tianjin Medical University General Hospital, Tianjin 300052, China; 2 Tianjin Neurological Institute, Tianjin 300052, China; 3 Department of Oncology-Pathology, Karolinska Institutet, Karolinska University Hospital Solna, Solna 17164, Sweden

**Keywords:** branched chain amino acid transaminase 1, IDH1, glioma, prognosis

## Abstract

Previous studies have reported the association between branched-chain amino acid trasaminase1 (BCAT1) and IDH1 wild-type gliomas. Nonetheless, as a promising target for treatment of primary glioblastoma, comprehensive reports on BCAT1 in gliomas are still lacking. In the present study, we accessed glioma patient cohorts and tissue microarray to evaluate the expression pattern of BCAT1 for determining its prognostic value and its relationship with IDH1 mutation status. Furthermore, we explored the potential regulatory mechanism of BCAT1 in gliomas by comparing the BCAT1 mRNA expression pattern with selected tumor biological signatures. The results showed that BCAT1 is highly expressed in GBM versus lower grade gliomas and could represent the poor survival of IDH1 wild-type gliomas. Moreover, BCAT1 is an independent prognostic factor for glioma patients, high BCAT1 expression is related to unfavorable clinical parameters including older age, IDH wildtype, no 1p/19q codeletion, ATRX wildtype and MGMT unmethylated. Additionally, BCAT1 correlated with apoptosis, hypoxia and angiogenesis processes in gliomas and high expression of BCAT1 revealed higher glycolysis level and increased immunosuppressive status in tumor progression. We concluded that BCAT1 is a strong prognostic factor for glioma patients and involved in the malignant progression of IDH1 wild-type gliomas.

## INTRODUCTION

Glioblastoma (GBM) is the most common malignancy in the adult central nervous system (CNS) and presents aggressive behavior and poor prognosis. Despite standard therapies, including tumor resection, concomitant radiotherapy, or adjuvant chemotherapy plus temozolomide, the outcome of patients is still very limited with a five-year overall survival of only 5.6% [1, 2]. Recent molecular analyses of glioma samples based on large-scale cohorts provide new inspiration for therapeutic development and clinical management [3, 4]. In 2016, the revision of the WHO classification of CNS tumors has highlighted the importance of the IDH1 or IDH2 mutation and co-deletion of chromosomal arms 1p and 19q for the diagnosis of gliomas [5]. This breaks with the old principle of diagnosis based entirely on phenotype by incorporating both histological and genetic alternations into the definition of new entities for the first time [6, 7].

Based on isocitrate dehydrogenase (IDH) 1/2 mutation status, glioblastomas can also be defined as primary (IDH1/2 wildtype), which originate de novo and secondary (IDH1/2 mutant), which evolve from lower grade gliomas (accounting for 80% IDH1/2 mutant cases) [8]. Compared to IDH1/2 mutant gliomas, the IDH wild-type gliomas represent the most devastating subgroups with heterogeneous genetic background and poor outcome [9–11]. However, understanding of the tumoral metabolism behind the development and progression of IDH wild-type gliomas remains lacking and new prognostic biomarkers and effective therapeutic targets for GBM still need to be identified.

Branched-chain amino acid transaminase 1 (BCAT1) is a cytosolic enzyme that catalyzes the transformation of branched-chain L-amino acids (BCAA) into branched-chain α-ketoacids (BCKA), with concomitant conversion of α-KG to glutamate [12, 13]. Emerging evidence suggests that BCAT1 plays a vital role in the progression of many cancers [14–17], especially highlighting the tight connection between BCAT1 level and IDH1 mutation status [18]. For glioma, it is indicated that loss of BCAT1 is a sensitive marker for IDH-mutant diffuse gliomas and that decreased expression of BCAT1 correlates with improved patient survival in IDH wild-type gliomas. ®-2-hydroxyglutarate, produced by IDH1/2 mutants, can inhibit the BCAT transaminases thus increases tumoral reliance on glutaminase for glutamate and glutathione synthesis [19, 20]. Suppression of BCAT1 in glioma cell lines blocked the excretion of glutamate and led to reduced proliferation and invasiveness *in vitro*, as well as significant decreases in tumor growth in a glioblastoma xenograft model [19, 21]. These studies indicate that BCAT1 is a biomarker for IDH1 wild-type gliomas and is essential for tumor metabolism to maintain an aggressive phenotype. Nonetheless, as a promising target in primary glioblastoma, comprehensive reports on the relationship between BCAT1 gene expression and clinical outcome or molecular features in glioma are still required.

In this study, we collected clinical and transcriptome (RNA-seq) data from The Cancer Genome Atlas (TCGA) and Chinese Glioma Genome Atlas (CGGA) databases, including 1395 glioma samples. Based on the large-scale sample collection, we analyzed the gene expression pattern of BCAT1 in glioma tissues as well as its relationship with IDH1 status and other clinical features. Furthermore, to evaluate potential biological processes that BCAT1 involves in the progression of glioma, we performed a pilot study where we examined the coexpression pattern of BCAT1 with different functional gene subgroups. This is the first comprehensive study to characterize BCAT1 expression in all grades of glioma molecularly and clinically and may provide bases for further evaluation of the roles BCAT1 in glioblastoma progression.

## RESULTS

### BCAT1 is highly expressed in GBM versus lower grade gliomas

**Figure 1:**
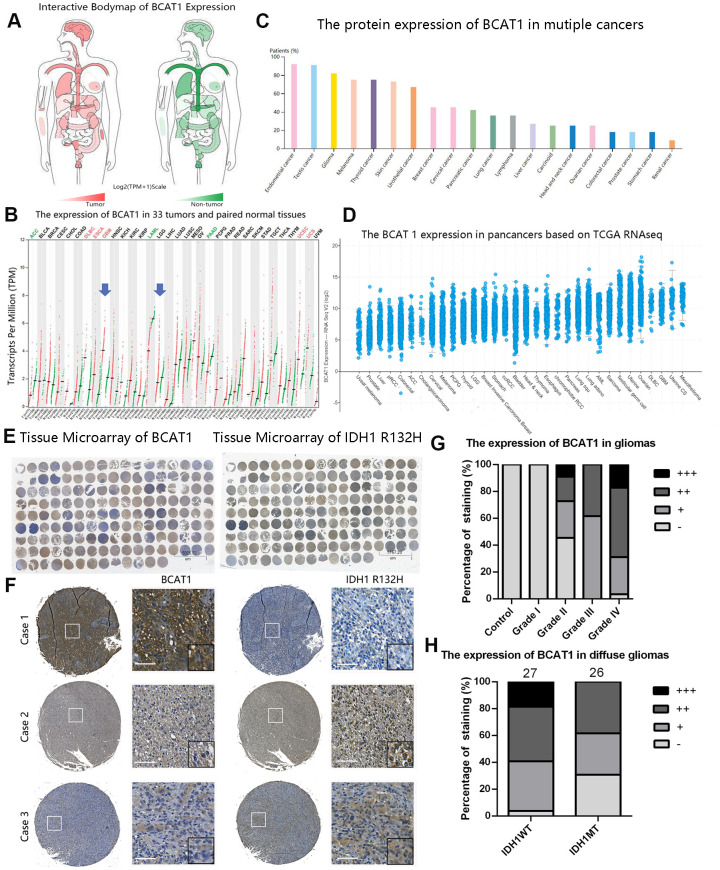
**The expression pattern of BCAT1 in pan-cancer and gliomas.** (**A**, **B**) The mRNA expression profiles of BCAT1 in 33 tumors and paired normal tissues were shown in bodymap and dot plot. (**C**) The expression pattern of BCAT1 protein in multiple cancer tissues from the Human Protein Atlas. (**D**) The BCAT1 expression in multiple cancer tissues from TCGA RNAseq. (**E**) The entire immunohistochemical staining of BCAT1 and IDH1 R132H in tissue microarray. (**F**) Representative images of BCAT1 and IDH1 R132H staining in three cases of glioma patients. (Case1: GBM, WHO Grade IV; Case2: Anaplastic oligodendroglioma, WHO Grade III; Case3: GBM, WHO Grade IV) (Scale: 100um) (**G**) Quantitative analysis of BCAT1 immunohistochemical staining in different grades of gliomas. (**H**) Quantitative analysis of IDH1 R132H immunohistochemical staining in IDH1 wild-type and mutant diffuse gliomas.

To evaluate the general expression pattern of BCAT1 in gliomas, we compared the mRNA and protein levels of BCAT1 between different tumors, grades, histological and molecular subtypes through several online databases. The mRNA expression of BACT1 in 33 types of tumor tissues (see the extension of tumor abbreviations in Supplementary Table 1) and its paired control tissues were shown in Figure 1A, 1B. Compared to other tumors, glioblastoma (GBM) owns one of the highest levels of BCAT1 expression. Notably, the mRNA level of BCAT1 in GBM is significantly higher than that in LGG. The Human Protein Atlas and TCGA databases also confirmed that gliomas own a high expression of BCAT1 among pan-cancers (Figure 1C, 1D). Collectively, these data indicate that BCAT1 is enriched in glioma tissues and upregulated in GBMs. Furthermore, we took advantage of clinical human glioma samples to detect the protein pattern of BCAT1 and IDH1 R132H in glioma tissues (Figure 1E, 1F). The results showed that BCAT1 is increasingly expressed in glioma tissues corresponding to increasing tumor grade (Figure 1G), and compared to IDH1 wild-type diffuse glioma patients (Figure 1H), the patients that had gained IDH1 R132H mutation showed a lower percentage of tumor cells with detectable BCAT1 expression. In addition, the data from GSE16011, GSE4290, REMBRANDT, TCGA and CGGA datasets reconfirmed our previous findings and detailed that BCAT1 is preferentially expressed in classical and mesenchymal subtype and less expressed in proneural subtype glioblastoma (Supplementary Figure 1).

### High BCAT1 expression is coupled with the poor survival of IDH1 wild-type gliomas

**Figure 2:**
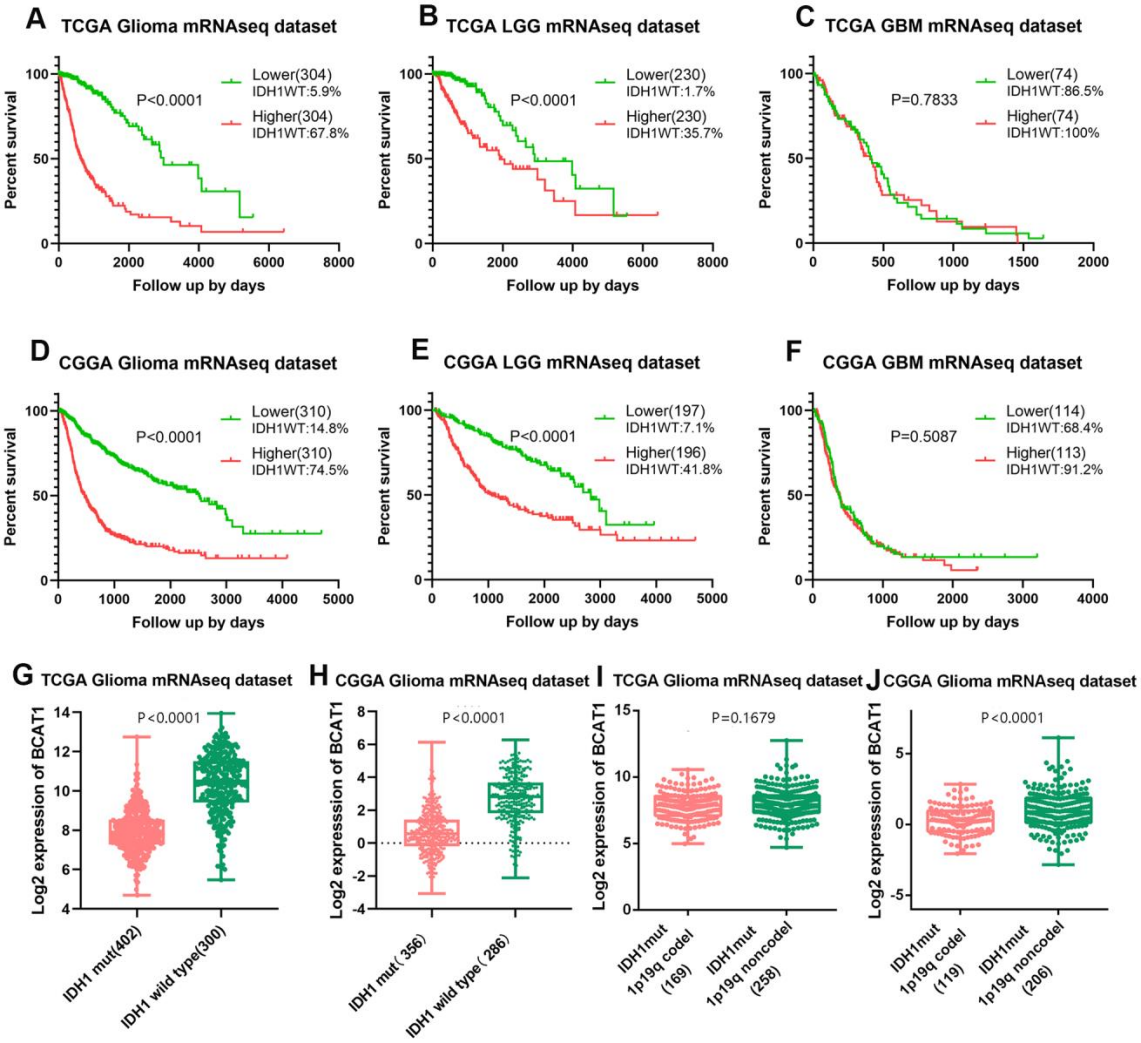
**High BCAT1 expression represents poor survival of IDH1 wild-type glioma patients.** Representative survival plots of BCAT1 in glioma patients with IDH wild-type proportion attached from TCGA (**A**–**C**) and CGGA (**D**–**F**) datasets. (**G**, **H**) Boxplots showing the distribution of BCAT1 expression in glioma patients according to IDH1 status from TCGA and CGGA datasets. (**I**, **J**) Boxplots showing the distribution of BCAT1 expression in IDH1 mutant, 1p19q codeleted glioma patients and IDH1 mutant, 1p19q non codeleted glioma patients from TCGA and CGGA datasets.

To determine the prognostic value of BCAT1 gene expression and its association with IDH1 status in glioma patients, Kaplan-Meier (K-M) survival curves with known IDH1 wildtype ratio was performed with data from the TCGA and CCGA RNA sequencing datasets. The results showed that the overall survival (OS) time of glioma patients with higher BCAT1 expression (TCGA, IDH1WT:67.8%; CGGA, IDH1WT:74.5%) is shorter than patients with lower BCAT1 expression (TCGA, IDH1WT:5.9%; CGGA, IDH1WT:14.8%) (Figure 2A, 2D). Moreover, in LGG, higher BCAT1 expression (TCGA, IDH1WT:35.7%; CGGA, IDH1WT:41.8%) is also connected with a worse overall survival time than lower BCAT1 expression (TCGA, IDH1WT:1.7%; CGGA, IDH1WT:7.1%) (Figure 2B, 2E), while in GBM, there is no significant difference between BCAT1 higher (TCGA, IDH1WT:100%; CGGA, IDH1WT:91.2%) and lower groups (TCGA, IDH1WT:86.5%; CGGA, IDH1WT:68.4%) (Figure 2C, 2F). Moreover, we found that IDH1 wild-type gliomas showed a higher expression of BCAT1 than mutant types in both TCGA and CGGA datasets (Figure 2G, 2H), and further internal comparison of IDH1 mutant gliomas showed that 1p19q codeleted IDH1 mutant gliomas owns a lower expression than non codeleted 1p19q forms with a significant difference in the CGGA dataset (Figure 2I, 2J). Those results implied that BCAT1 contributes to the poor survival of IDH1 wild-type glioma patients.

### BCAT1 is an independent prognostic factor related to higher tumor malignancy in glioma patients

To further delineate the impact of BCAT1 gene expression on the patients’ treatment response, we conducted an overview of BCAT1 expression together with several genetic alterations which are known to be related with progression of glioma. In the TCGA dataset, patient groups of younger age, IDH mutation, 1p/19q deletion, ATRX mutation and MGMT methylation, are associated with lower BCAT1 expression (P < 0.05). Treatment with radiotherapy and additional pharmaceutical therapy was also more likely occurred in BCAT1 low expression patients (P < 0.05) (Table 1). The results are consistent with CGGA dataset (Table 2), in which low expression of BCAT1 is related with younger age, IDH mutation, 1p/19q codeletion and chemoradiotherapy. Besides, patients with high BCAT1 expression are also more likely to develop tumor recurrence. The result of COX regression analysis (Table 3) in the CGGA cohort indicates IDH mutation status, 1p/19q codeletion status and the expression of BCAT1 can be considered as independent clinical prognostic factors. We obtained the same result from COX regression analysis in the TCGA cohort. According to all of the above evidences, high BCAT1 expression is associated with several clinicopathological parameters and could serve as an independent prognostic predictor for glioma patients.

**Table 1:** The association between BCAT1 expression and clinical features in TCGA dataset.

	**BCAT1 LOW**	**BCAT1 HIGH**	**Test value**	**P value**
**Age (≤60/>60)**	71/6	56/21	10.105	0.001
**Gender (M/F)**	43/34	37/40	0.936	0.333
**Grade (LGG/GBM)**	76/1	59/18	17.351	0.000
**IDH Mutation (Y/N)**	4/73	49/28	58.257	0.000
**1p/19q deletion (Y/N)**	51/26	67/10	9.281	0.002
**MGMT Methylation (Y/N)**	8/69	34/43	22.131	0.000
**ATRX Mutation (Y/N)**	32/45	58/19	18.074	0.000
**Radiotherapy (Y/N)**	28/49	19/58	2.480	0.115
**Additional pharmaceutical therapy (Y/N)**	37/40	34/43	0.235	0.628

**Table 2:** The association between BCAT1 expression and clinical features in CGGA dataset.

	**BCAT1 LOW**	**BCAT1 HIGH**	**Test value**	**P value**
**Age (≤60/>60)**	235/11	211/36	14.587	0.000
**Gender (M/F)**	141/105	138/109	0.105	0.746
**Grade**			-10.205	0.000
**2**	97	34		
**3**	116	67		
**4**	33	146		
**IDH Mutation (Y/N)**	213/33	62/185	188.893	0.000
**1p19q deletion (Y/N)**	96/150	9/238	92.043	0.000
**PRS_type (Primary/Recurrent)**	163/83	139/108	5.178	0.023
**Radiotherapy (Y/N)**	192/54	212/35	5.044	0.025
**Chemotherapy (Y/N)**	167/79	201/46	11.851	0.001

**Table 3:** Multivariate survival analysis of overall survival probabilities with respect to BCAT1 expression based on TCGA and CGGA datasets.

**Variables**	**Univariate analysis**			**Multivariate analysis**		
	**HR**	**95%CI**	**P value**	**HR**	**95%CI**	**P value**
**TCGA (n=620)**						
Age	5.731	4.248-7.732	0.000	2.658	1.912-3.697	0.000
Gender	0.889	0.673-1.173	0.405	-	-	-
IDH status	0.102	0.075-0.138	0.000	0.251	0.148-0.425	0.000
1p19q	0.248	0.158-0.390	0.000	0.482	0.265-0.875	0.016
ATRX	0.460	0.333-0.636	0.000	0.692	0.437-1.095	0.116
MGMT	0.305	0.230-0.404	0.000	1.026	0.734-1.436	0.879
BCAT1	4.506	3.283-6.184	0.000	1.626	1.104-2.396	0.014
**CGGA (n=465)**						
Age	2.079	1.447-2.989	0.000	1.215	0.833-1.772	0.312
Gender	1.101	0.862-1.407	0.442	-	-	-
Radiotherapy	1.379	0.970-1.962	0.073	-	-	-
Chemotherapy	1.606	1.177-2.192	0.003	1.382	1.008-1.895	0.045
IDH status	0.274	0.213-0.353	0.000	0.503	0.365-0.694	0.000
1p19q	0.283	0.191-0.420	0.000	0.545	0.354-0.841	0.006
BCAT1	3.513	2.687-4.593	0.000	1.895	1.355-2.649	0.000

### BCAT1 correlates with apoptosis, hypoxia, and angiogenesis processes in gliomas

**Figure 3:**
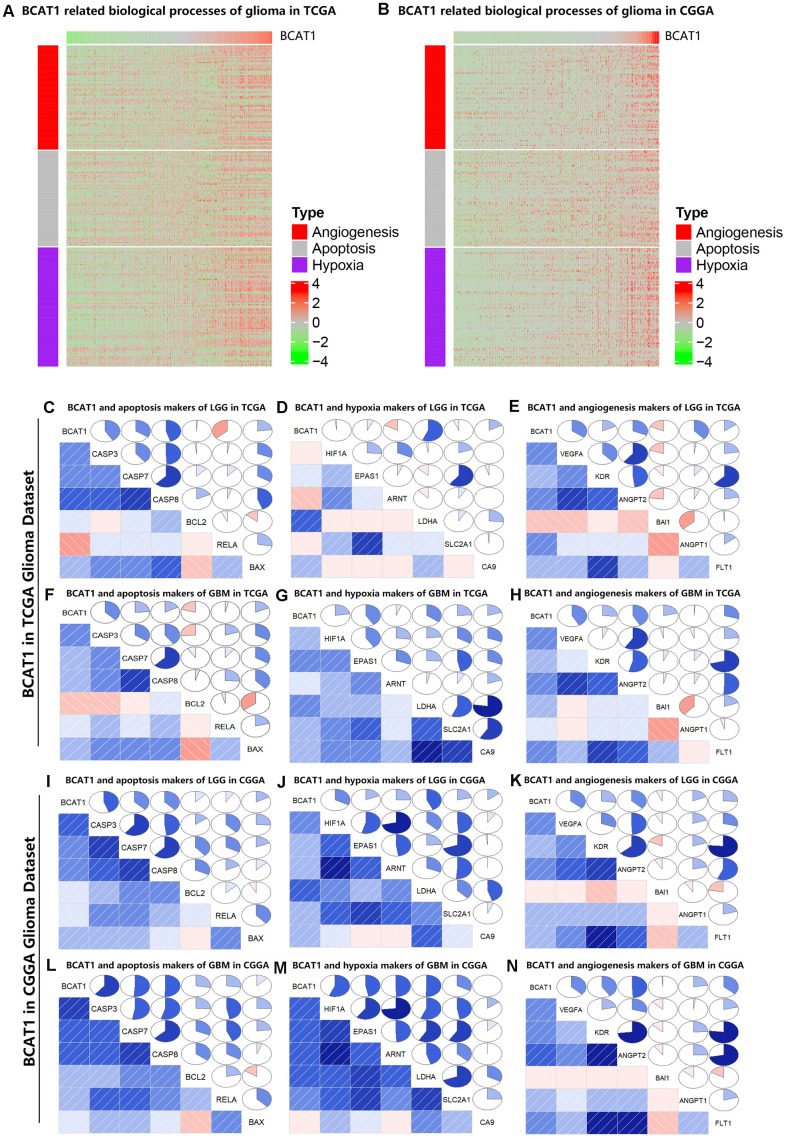
**BCAT1 is correlated with apoptosis, hypoxia and angiogenesis processes in gliomas.** (**A**, **B**) Heatmaps showing the expression patterns of angiogenesis, apoptosis and hypoxia markers in glioma patients according to BCAT1 expression based on TCGA and CGGA datasets. (**C**–**E**) The relation between BCAT1 expression and apoptosis, hypoxia and angiogenesis markers in LGG of TCGA dataset. (**F**–**H**) The relation between BCAT1 expression and apoptosis, hypoxia and angiogenesis markers in GBM of TCGA dataset. (**I**–**K**) The relation between BCAT1 and apoptosis, hypoxia and angiogenesis markers in LGG of CGGA dataset. (**L**–**N**) The relation between BCAT1 and apoptosis, hypoxia and angiogenesis markers in GBM of CGGA dataset.

To illuminate the biological features of glioma with different BCAT1 expression, we examined the correlation of BCAT1 expression with angiogenesis, apoptosis and hypoxia marker genes to evaluate the relationship between BCAT1 expression and tumor microenvironment. As shown in functional heatmap analyses, glioma-derived BCAT1 expression was positively correlated with biomarker gene expression of apoptosis, hypoxia and angiogenesis in both the TCGA and CGGA datasets (Figure 3A, 3B). Similarly, Pearson correlation analysis was performed to retest the correlation of interest gene sets with BCAT1 expression in LGG and GBM based on TCGA and CGGA datasets. After screening several apoptosis markers including CASP3, CASP7, CASP8, BCL2, RELA and BAX; hypoxia markers including HIF1A, EPAS1, ARNT, LDHA, SLC2A1 and CA9; angiogenesis markers including VEGFA, KDR, ANGPT2, BAI1, ANGPT1 and FLT1 as shown by correlograms, the results were consistent with the heatmap analysis. BCAT1 expression was positively correlated with apoptosis, hypoxia and angiogenesis markers (Figure 3C–3N). Interestingly, it seems BCAT1 is tighter associated with these genes in GBM than in LGG (Figure 3C–3N). These results suggested that BCAT1 is associated with the apoptosis, hypoxia and angiogenesis processes in gliomas, especially in GBM.

### High BCAT1 expression is correlated with higher glycolysis level in gliomas

**Figure 4:**
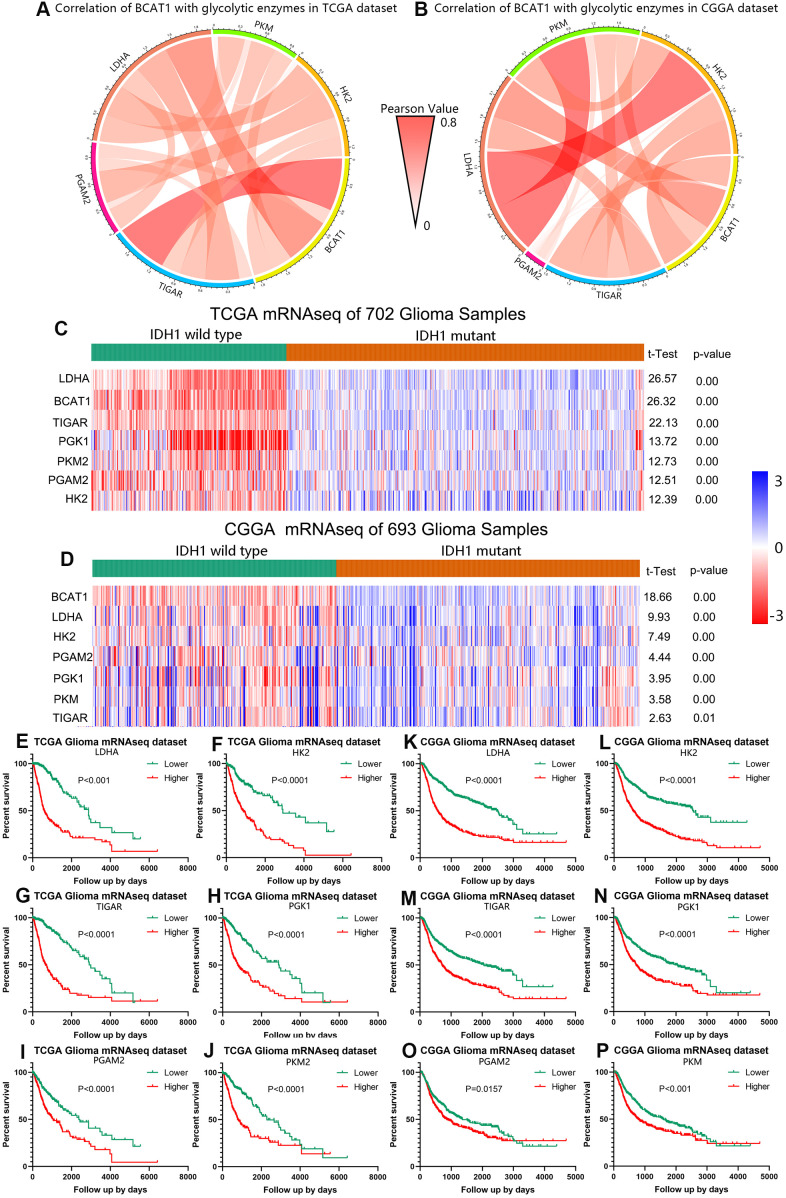
**BCAT1 is correlated with glycolysis process in gliomas.** (**A**, **B**) The correlation of BCAT1 with glycolytic enzymes in TCGA and CGGA datasets. (**C**, **D**) Heatmap showing the mRNA expression of BCAT1 and related glycolytic enzymes in IDH1 wild-type and mutant gliomas based on TCGA and CGGA datasets. (**E**–**J**) Survival plots for BCAT1 correlated glycolytic enzymes in TCGA glioma patients. (**K**–**P**) Survival plots for BCAT1 correlated glycolytic enzymes in CGGA glioma patients.

The tumor metabolic phenotype is characterized by preferential dependence on glycolysis, and glycolytic enzymes are also known to be associated with the worse or better prognosis in various cancers. To evaluate the relationship between BCAT1 and glycolysis in gliomas, we firstly performed the Pearson correlation analysis using both TCGA and CGGA datasets. After screening several glycolytic enzymes including LDHA, PGAM2, TIGAR, PKM, HK2, we found that BCAT1 expression was positively correlated with glycolysis level as shown by Circos plots (Figure 4A, 4B). Moreover, IDH1 wild-type gliomas displayed a significantly increased expression of glycolytic enzyme genes (LDHA, HK2, PGAM2, PGK1, PKM, TIGAR) compared to IDH1-mutant gliomas as shown in heatmaps (Figure 4C, 4D). Finally, we evaluated the prognostic value of these glycolytic genes based on the two glioma datasets to determine their effect on glioma patients’ survival. As shown in Figure 4E–4J, higher expression of glycolytic enzyme genes predicted worse overall survival (OS) in glioma based on the TCGA dataset. Similarly, a strong correlation between higher expression of glycolytic enzyme genes and shorter patient OS was also observed in the CGGA dataset (Figure 4K–4P). These findings indicate that BCAT1 might synergize with glycolytic enzymes in the worse outcome of IDH1 wild-type gliomas.

### High BCAT1 expression is accompanied by increased immunosuppressive status in the tumor

**Figure 5:**
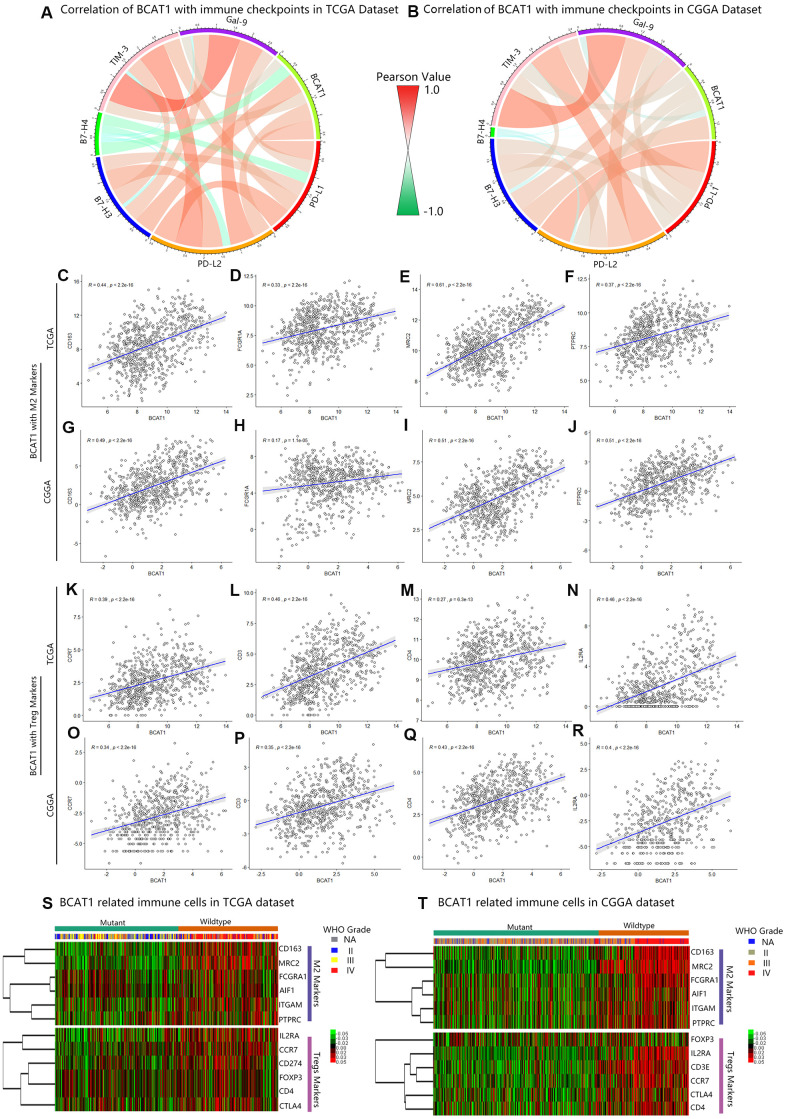
**BCAT1 is correlated with immunosuppressive status in gliomas.** (**A**, **B**) The correlation between BCAT1 and immune checkpoints in the TCGA and CGGA datasets. (**C**–**F**) The correlation between BCAT1 and M2 markers in the TCGA dataset and (**G**–**J**) CGGA dataset. (**K**–**N**) The correlation between BCAT1 and Treg markers in the TCGA dataset and (**O**–**R**) CGGA dataset. (**S**, **T**) Heatmap showing the mRNA expression pattern of BCAT1 related immune cell markers in the TCGA and CGGA datasets, shown respectively for IDH1 mutant and wild-type gliomas and for glioma grades.

The immunological tumor microenvironment of diffuse gliomas differs in regards to IDH mutation status. IDH1 wild-type gliomas display a more immunosuppressive tumor microenvironment including a more prominent immune cell infiltration and higher PD-L1 expression [22]. To further explore the role of BCAT1 in the immunosuppressive status of gliomas, Circos plots were performed based on the correlation between BCAT1 and six immune checkpoints (PD-L1, PD-L2, B7H3, B7H4, TIM-3, Gal-9). The results showed that BCAT1 synergized well with other immune checkpoint molecules in gliomas, which were observed in both TCGA and CGGA datasets (Figure 5A, 5B). The Pearson correlation analysis further showed that BCAT1 was positively associated with M2 macrophage markers (CD163, FCGR1A, MRC2, PTPRC) and Treg markers (CCR7, CD3, CD4, IL2RA) in both TCGA (Figure 5C–5J) and CGGA (Figure 5K–5R) datasets. Then, mutual relationship analysis of BCAT1 related immune cells was further conducted to identify their clusters according to IDH status and tumor grades. The results demonstrated that M2 macrophages and Tregs were preferentially involved in IDH1 wild-type and higher grades gliomas (Figure 5S, 5T). Overall, these data suggest BCAT1 is correlated with the immunosuppressive signature of IDH1 wild-type gliomas.

## DISCUSSION

Despite IDH1 is a powerful prognostic marker to distinguish lower grade gliomas and secondary glioblastoma from primary glioblastoma [23–25], its potential role as a therapeutic target for gliomas has not yet been determined [26] and further understanding of IDH1 in tumor metabolism is essential [27, 28]. Recent studies have focused on the crosstalk between BCATs and IDH1 in the catabolism of glioblastoma [12, 29, 30]. It was indicated that IDH1 mutation is associated with changes in cellular metabolism, which includes decreased branched-chain amino acid transaminase 1 (BCAT1) activity [31, 32]. Specifically, BCAT1 expression is dependent on the concentration of α-ketoglutarate (α-KG) substrate and could be suppressed by downregulation of IDH1 in glioblastoma cell lines or overexpression of mutant IDH1 in immortalized human astrocytes [19]. In addition, BCAT1 knockdown can strongly restrict tumor growth and progression in glioblastoma [19]. However, the associated molecular mechanisms of BCAT1 in glioma remain poorly understood, and thus more extensive research in this area could provide insight towards improved diagnosis and treatment.

In this study, we evaluated the expression of BCAT1 in gliomas and its potential involvement in tumor biological processes. From our results, BCAT1 is higher expressed in glioblastoma than in lower grade gliomas and higher in astrocytoma than in oligodendroglioma as well as higher in classical and mesenchymal subtypes than in proneural subtype glioblastoma. These findings imply that BCAT1 possibly influences overall survival time and other clinical features in glioma patients, which was validated in both TCGA and CGGA datasets. Moreover, the association between BCAT1 and IDH1 status was also confirmed, as previously reported, high expression of BCAT1 is observed in IDH1 wild-type gliomas but is also preferentially expressed in non 1p19q co-deleted gliomas. Besides, high BCAT1 expression represents poor survival of IDH1 wild-type gliomas. These results indicated a malignant biological property for BCAT1 in IDH wild-type gliomas. Emerging data has demonstrated a critical role for BCAT1 in glioblastoma growth. To further demonstrate the mechanisms underlying BCAT1 regulated IDH1 wild-type gliomas. we explored the association between BCAT1 and apoptosis, hypoxia and angiogenesis metagenes, BCAT1 expression was positively related with apoptosis, hypoxia and angiogenesis processes in gliomas especially in GBM. Furthermore, as a key enzyme in BCAAs and energy metabolism, its association with glycolysis was also identified. BCAT1 expression was positively correlated with glycolytic enzymes, which are enriched in IDH1 wild-type gliomas and related to poor overall survival of glioma patients.

Tumor-infiltrating Treg cells and M2 cells are a major cause of poor clinical outcome in many cancers. Increasing evidence suggests that accumulation of Treg and M2 macrophage cells can suppress antitumor immunity through various cellular and humoral mechanisms including expressing immune-checkpoint molecules [33–37]. In glioma, human IDH1-mutant gliomas have less infiltrating immune cells than IDH1 wild-type gliomas [22]. From our results, BCAT1 is positively related to immune checkpoints and expressed synergistically with other checkpoint members. Meanwhile, high expression of BCAT1 is also linked with a high percentage of M2 and Treg cell infiltration, which are more frequently occurring in IDH wild-type gliomas. These findings indicate that BCAT1 may also involve in modulating the tumor-induced immune response to IDH1 wild-type gliomas.

## CONCLUSIONS

To conclude, our work highlights that high expression of BCAT1 is a sensitive marker for predicting poor prognosis of IDH1 wild-type glioma patients. BCAT1 is enriched in IDH1 wild-type gliomas and may relate to apoptosis, hypoxia and angiogenesis processes in tumor progression. Moreover, BCAT1 is also involved in the glycolytic metabolism and immune suppression of IDH1 wild-type gliomas, suggesting multiple roles that BCAT1 exerts in the malignant transformation of glioblastoma. Therefore, BCAT1 represents a potential therapeutic target and useful prognostic factor for glioblastoma patients.

## MATERIALS AND METHODS

### Databases

There were six glioma mRNA datasets used in this study. The RNA sequencing expression and clinical information of 702 samples were downloaded from The Cancer Genome Atlas (TCGA) database (https://www.cancer.gov/). The Chinese Genome Atlas (CGGA) RNA sequencing dataset and clinical characteristics of 693 samples were obtained from the publicly available website (http://www.cgga.org.cn/). The GSE16011, GSE4290 and REMBRANDT (GSE108474) datasets were downloaded from Gene Expression Omnibus (GEO, https://www.ncbi.nlm.nih.gov/geo/). Data on immunohistochemical detection of BCAT1 in gliomas and other tumor samples were obtained from The Human Protein Atlas (https://www.proteinatlas.org/).

### Tissue microarray immunohistochemistry

The clinical information and the procedure for immunohistochemical staining of human glioma samples have been described previously [38, 39].

### Bioinformatic analysis

The gene expression of BCAT1 in tumor and non-tumor tissues was obtained and analysis was performed by Gene Expression Profiling Interactive Analysis (GEPIA, http://gepia.cancer-pku.cn/). The mRNA expression of BCAT1 in different glioma grades, subtypes and other conditions was calculated by GraphPad Prism 8. The markers for apoptosis, hypoxia and angiogenesis were selected based on signature profiles from CancerSEA. The heatmaps revealed the mRNA expression of BCAT1 and other genes of apoptosis, hypoxia and angiogenesis in the TCGA and CGGA datasets R programing language using “complexheatmap” package. The correction analysis between BCAT1 and other genes of apoptosis, hypoxia and angiogenesis in the TCGA and CGGA datasets was performed through “corrplot” package. Circos plots revealing the correlation of BCAT1 with glycolytic enzymes and immune checkpoints were conducted by R language using “circlize” package. The Pearson correction analysis between BCAT1 and immune cell (M2, Treg) markers was conducted by R language using “ggpubr” package.

### Statistical analysis

Kaplan-Meier survival analysis manifesting the influence of the expression of BCAT1 and other related genes on overall survival time and median value was used as the cutoff to define high and low expression group. Univariate and multivariate COX regression analyzed by SPSS 21.0 including age, gender, IDH status, 1p19q, BCAT1, radiotherapy and chemotherapy were used to evaluate the impact of above factors on the prognostic value in glioma patients. Chi-square test and rank sum test were used to verify if the expression of BCAT1 is distinct in different groups of age, gender, grade, IDH status, 1p19q, MGMT, ATRX, radiotherapy and chemotherapy. One-way ANOVA was used to test for differences among at least 3 groups. The t test was used to determine differences in each double group comparison. In all statistical analyses, p value less than 0.05 was considered to be statistically significant.

## Supplementary Material

Supplementary Figure 1

Supplementary Table 1
