# Supplementary material for: Enrichment of branched chain amino acid transaminase 1 correlates with multiple biological processes and contributes to poor survival of IDH1 wild-type gliomas
Source: Aging (Albany NY). 2021 Jan 20;13(3):3645–60. doi: 10.18632/aging.202328 (PMC7906175; doi:10.18632/aging.202328)
Supplement: Supplementary Figure 1 [file aging-13-202328-s001.pdf]

SUPPLEMENTARY FIGURE

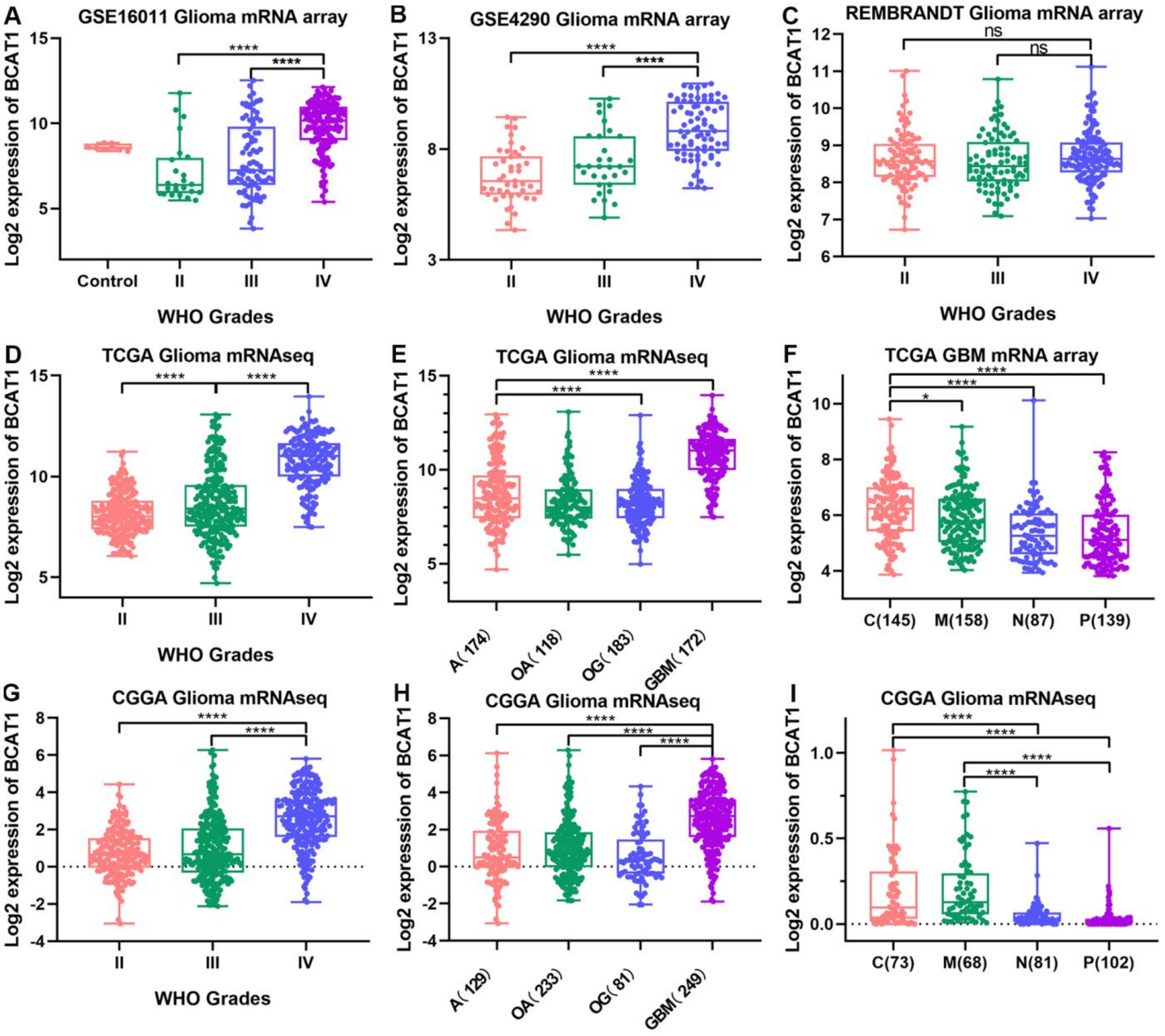

Supplementary Figure 1. (**A**–**C**) The mRNA expression of BCAT1 in different grades of gliomas according to GSE16011, GSE4290 and REMBRANDT datasets. (**D**–**I**) The mRNA expression profiles of BCAT1 in different grades, histological types and molecular subtypes of gliomas according to TCGA and CGGA datasets. (Abbreviations: A: astrocytoma, OA: oligoastrocytoma, OG: oligodendroglioma, GBM: glioblastoma; C: classical, M: mesenchymal, N: neural, P: proneural) (****:P<0.0001, ***:P<0.001, **:P<0.01, *:P<0.05, ns: no significance).
